# Study on the Safety of the New Radial Artery Hemostasis Device

**DOI:** 10.1155/2022/2345584

**Published:** 2022-04-05

**Authors:** Baofeng Wu, Ruixin Zhang, Chendi Liang, Chengjie Zhang, Gang Qin

**Affiliations:** ^1^Department of Cardiology, First Hospital of Shanxi Medical University, No. 85, JieFang South Road, Yingze District, Taiyuan, Shanxi 030001, China; ^2^First Clinical Medical College, Shanxi Medical University, No. 85, JieFang South Road, Yingze District, Taiyuan, Shanxi 030001, China

## Abstract

**Objective:**

At present, the use of particular radial hemostatic devices after coronary angiography (CAG) or percutaneous coronary intervention (PCI) has become the primary method of hemostasis. Most control studies are based on the products already on the market, while only a few studies are on the new hemostatic devices. The aim of this study is to compare a new radial artery hemostasis device which is transformed based on the invention patent (Application number: CN201510275446) with TR Band (Terumo Medical) to evaluate its clinical effects.

**Methods:**

In a prospective randomized clinical trial, 60 patients after CAG or PCI were randomly divided into two groups, patients in the trial group (CD group) using a new radial artery hemostasis device to stop bleeding and the control group (TR group) using the TR Band. The method is to collect relevant data of the two groups and compare the differences in hemostasis, local complications, and patient discomfort between the two groups.

**Results:**

The hemostatic devices in both groups achieved adequate hemostasis, and there was no failure to stop bleeding. The new radial artery hemostasis device was better than the TR band in pain and swelling (*P* < 0.05). There were no significant differences in bleeding, hematoma, ecchymosis, skin damage, and local infection between the two groups (*P* > 0.05).

**Conclusions:**

The sample of the new radial artery hemostasis device can stop bleeding effectively at the puncture site after CAG or PCI and is not inferior to the TR Band balloon hemostatic device in safety and is better in comfort.

## 1. Background

The diagnosis and treatment of coronary artery disease (CAD) are mainly achieved through CAG and PCI, and transfemoral access (TFA) is a choice at the beginning. However, with the increase in angiography and interventional procedures, the complications of TFA are increasing gradually, and the most common ones are arteriovenous fistula, pseudoaneurysm, and arterial dissection [1]. Besides, the hematoma is more likely to occur due to compression techniques and other reasons, so TFA is often suitable for some high-risk or complex coronary intervention operations. Some clinical studies have also been carried out on transulnar access (TUA). However, the ulnar artery is deep and has a higher bifurcation rate, and it often requires more puncturing times. Besides, it is also accompanied by the ulnar nerve [2], so it is generally not the first choice, but TUA is an alternative for patients who are unsuitable for TFA and have radial artery hypoplasia or malformation [3]. Transradial access (TRA) is preferred for CAG and PCI due to its superficial location, fewer associated complications, easier hemostasis, higher comfort, and patients who do not need braking and more special treatment after the operation [4, 5]. Many studies have shown that TRA is superior to TFA in reducing clinical adverse events and complications at the puncture site, regardless of routine surgery or treating complex lesions [6, 7].

Although TRA is often chosen, it also has certain complications, such as bleeding and hematoma at the puncture site, radial artery spasm, radial artery occlusion (RAO), even radial artery lacerations, pseudoaneurysm, and osteofascial compartment syndrome [8]. The application of radial hemostatic devices is widely used, especially in TR Band. Many studies have shown that TR Band has advantages in clinical use, such as simple operation, good hemostatic effect, and low complication rates [9–11]. In this study, a sample of a new radial artery hemostatic device based on a patent was evaluated by comparing its effectiveness, safety, and comfort with the TR Band and analyzing clinical data to provide new ideas and directions for the development of hemostats in the future.

## 2. Methods

First, we transformed the radial artery hemostatic device involved in an invention patent (Application number: CN201510275446). According to the design points of the patent, we completed the revised design (Supplementary Figure 1) and sample production of the new radial artery hemostatic device through the following stages. The first stage: according to the patent instrument, the preliminary design of the sample was completed, the compression subject adopted the design of the airbag bar, and the wristband was made of medical silica. The second stage: the compression subject and the wristband adopted a separate design, with groove and slits on the wristband for fixing the compression subject, and the wristband was fixed through buckles, so we obtained the sample that could be used in clinical trials (the wristband was provided by Jiangsu Daorong Electronic Technology Co. Ltd., and the compression subject was provided by Jiangsu Aeris Medical Technology Co. Ltd., all materials were sterilized with ethylene oxide and met conditions for the biological evaluation of medical devices) (Supplementary Figure 2). The third stage: completing the test of hemostatic pressure and obtaining the air volume-pressure curve by comparing it with TR Band to provide the reference for the air-filling volume of the sample (RPM Pressure Tester, Shanghai Ruiruo Measurement and Control Equipment Co., LTD) (Supplementary Figure 3).

### 2.1. Study Design and Population

**Figure 1:**
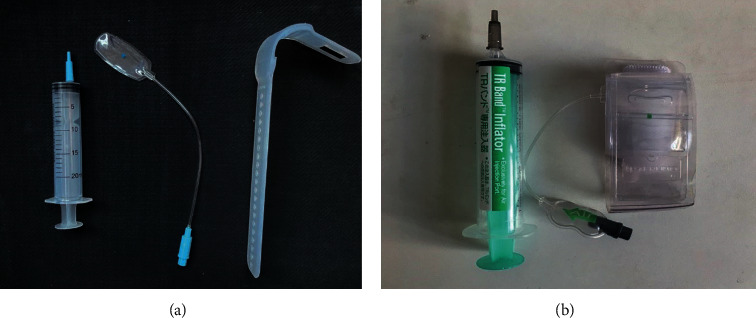
Study devices. The new radial artery hemostatic device which based on the patent design (a) and the Terumo TR Band™ (b).

This study was a noninferiority study. A total of 60 patients in our hospital who were prepared to undergo elective CAG or PCI from February 2021 to April 2021 were collected and randomly divided into average groups. Patients in the trial group (CD group) were treated with a new radial artery hemostatic device (Figure 1(a)), while the control group (TR Group) used TR Band (Figure 1(b)). The sample size was calculated according to the bleeding rate, and it was found that the bleeding rate fluctuated in different ranges after using TR Band, but most studies showed that the bleeding rate fluctuated around 5% after using TR Band [12–14]. Considering the differences in airbag filling, decompression time interval, and hemostatic compression time in various clinical studies, and based on the experience of our clinical center, we set the control group (TR Group) bleeding rate at 5%, the experimental group (CD Group) bleeding rate expectations for 10% (considering the operator learning curve), and the non-inferiority threshold was set at-15%, which allowed for the calculation of approximately 27 samples from each group, considering the 10% lost to follow-up rate, sample size determined for 60 cases finally. Due to this study's nature and time limit, recruits were kept small, and the study was not blinded because of the difference in hemostatic devices.

Inclusion criteria were as follows:

1. Patients 18 years or older who underwent coronary intervention for the first time
2. The right radial artery was the access, and the pulse was good
3. Patients were sanity and expressed clearly
4. No activity limitation of the right upper limb

Exclusion criteria were as follows:

1. Patients who underwent emergency surgery
2. The TRA was not successful
3. Patients who had skin damage and scarring at the wrist joint
4. Patients who had fractures of the wrist joint
5. Clinicians judged patients with a high risk of bleeding

This study was approved by the Ethics Committee of the First Hospital of Shanxi Medical University (Date of approval: 2021-01-11, Approval number: 2020K013, Supplementary File 4), and all the study subjects signed the informed consent.

### 2.2. Procedure

After the puncture site was determined, 1% lidocaine was used for local anesthesia. After the successful puncture, guidewire and 5 Fr sheath (Terumo Medical) were implanted, and heparin sodium was used for systemic heparinization (60–80 U/kg), verapamil 2.5 mg and nitroglycerin 200 *μ*g were used to relieve radial artery spasm. The same two experienced interventional cardiologists performed all the procedures.

### 2.3. Hemostasis Methods

The manufacturer of the TR Band did not specify when the device could be safely removed. In our clinical center, the operator withdrew the catheter sheath 3–5 cm after the operation, fixed the hemostatic device, and made the mark of Terumo on the support plate close to the ulnar side. Then, the operator used the supporting inflator to expand the balloon by injecting air into the compressed balloon until there was no bleeding (standard air injection volume 13 ml, maximum air injection volume 18 ml). Finally, the operator pulled out all the sheath and observed whether there was bleeding. If there was bleeding, inject 1–2 ml of air again until the bleeding stops [15]. If there were no special conditions, 2 ml of air was deflated every 1 hour after the operation, and the air was extracted entirely 5 hours later. Then, the compression device was removed, and the puncture site was covered with a sterile dressing. In order to avoid more operational errors, both the CD group and the TR Group adopted the same inflating and deflating protocol. However, for the CD group, the operation was different in some ways: before the sheath was removed, the airbag bar in its uninflated state should be put into the accommodating groove of the silicone wristband, so the mark point of the bar could be aligned with the puncture point, and the entire compression device was fixed on the wrist by adjusting the position of the buckle.

If bleeding occurred at the puncture site in both groups during decompression, the air should be reinjected until bleeding stops, and decompression should be extended for half an hour. Participants were trained and simulated before the operation, and two trained physicians performed all hemostasis procedures.

### 2.4. Endpoints

The study's primary endpoint was adequate hemostasis, defined as the use of other alternative hemostasis because bleeding cannot be stopped. The second endpoint was to observe local complications at the puncture site during the decompression process or after the removal of the hemostatic device, specifically including (1) bleeding: bleeding during decompression and requiring reinjection of air or a large amount of bleeding still required compression after the removal of the device; (2) hematoma: due to local injury, the dermal capillaries were damaged and hyperemia, fluid and cell components exudate, retention in the skin and subcutaneous tissue, small hematoma (diameter ≤2 cm) and large hematoma (diameter >2 cm); (3) ecchymosis: purplish-red blood spots with a diameter of more than 5 mm appear on the skin surface; (4) skin lesion: local skin rupture and blisters due to the compression of wristband; (5) local infection: redness, swelling, heat, pain, and even dysfunction occurred at the puncture site.

The visual analog scale (VAS) evaluated the patients' discomfort, including pain, numbness, and swelling. The degree of feeling was judged according to the score (“0”means no sensation; “1–3” means mild; “4–6” indicates moderate; “7–9” indicates severe; “10” means solid and unbearable feeling), and the patients can assess their subjective feelings. In order to evaluate the patency of the radial artery, plethysmography and pulse oxygen saturation were used for preliminary verification, which was more sensitive than the modified Allen test in the comparison of the collateral circulation of the hand [16]. Related observation indicators were collected before (0 0 h), postoperative 1 h, 6 h, 24 h.

### 2.5. Statistical Analysis

Statistical analysis was performed using the SPSS 23.0 statistical program (SPSS Inc, Chicago, Illinois). The measurement data were normally distributed and expressed as mean ± standard deviation. We used the Independent-Samples *T* test to compare the differences of each index among the groups. The categorical data were expressed as *n* (%), and the chi-square test (Fisher's exact test) was used to compare the differences of each index among the groups. The ranked data were expressed as *n* (%), and the Mann–Whitney *U* test was used to compare the differences of each index between groups. *P* < 0.05 was considered statistically significant.

## 3. Results

Baseline subject characteristics are shown in Table 1, there were no significant differences in age, gender, wrist circumference, body mass index, past history, family history, smoking history, platelet count, coagulation, creatinine, and regular use of antiplatelet drugs between the two groups (*P* > 0.05). There were no significant differences between the two groups in procedure characteristics such as sheath size, heparin used during the process, procedure time, and the percentage of CAG.

Patients in the CD group used a new radial artery hemostatic device, and patients in the TR group used the TR Band. Both groups achieved adequate hemostasis without failure to stop bleeding. The differences in bleeding rate (10.00% vs. 3.33%, *P*=0.612), ecchymosis (6.67% vs. 6.67%, *P*=1.000), hematoma (3.33% vs. 0, *P*=1.000), skin lesion, and local infection between the new radial compression device group and the TR Band group were not statistically significant (*P* > 0.05) (Table 2). It can be considered that there was no difference in the incidence of complications between the two devices.

**Figure 2:**
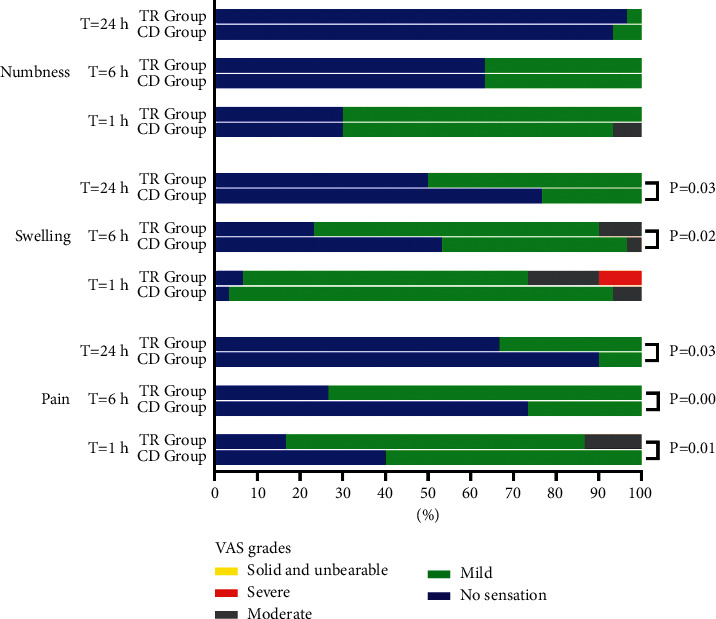
Comparison of subjective discomfort in two groups at different time points. The pain, numbness, and swelling were evaluated by VAS, and the scores were divided into different grades. Independent rank sum tests were used to compare the differences between the two groups. The vertical axis represents subjective discomfort at different time points in the two groups, and the horizontal axis represents the sum of percentages.

The results of the patients' subjective discomfort are shown in Figure 2. There was no statistical significance in the occurrence of numbness between the two groups at 1 h, 6 h, and 24 h after operation (*P* > 0.05). In the occurrence of swelling, there was a statistically significant difference between 6 h and 24 h postoperatively (*P* < 0.05). In terms of the occurrence of pain, there were statistically significant differences between the two groups at 1 h, 6 h, and 24 h after operation (*P* < 0.05).

The Independent-Samples *T* test is applied to compare the differences between the CD group and the TR group, and the results are shown in Table 3. There was no statistically significant difference in SpO_2_ and wrist circumference between the two groups at different time points (*P* > 0.05). There was no significant difference in the effects of the two devices on distal blood supply and wrist circumference. There were statistically significant differences in systolic blood pressure between the two groups at 1 h, 6 h, and 24 h after operation (*P* < 0.05). The difference in diastolic blood pressure at 6 h and 24 h after operation was statistically significant (*P* < 0.05), the new radial artery hemostatic device and TR Band showed different effects on blood pressure. The difference of pulse between the two groups at 1 h and 6 h after operation was statistically significant (*P* < 0.05).

## 4. Discussion

The incidence of coronary artery disease increases yearly, and the diagnosis and treatment are mainly made by coronary angiography and percutaneous coronary intervention. The access is initially chosen via the femoral artery, but it will increase the risk of hemostasis time and related complications after TFA, whether manual or mechanical hemostasis is used [17]. In contrast, the radial artery is currently the preferred access due to its superficial location and less variability while providing a dual blood supply to the hand with the ulnar artery. Compared with the TFA, the TRA reduces the risk of bleeding and vascular complications, improves safety, and reduces mortality and major adverse cardiovascular events in patients with coronary artery disease [18, 19]. According to the European Society of Cardiology guidelines, TRA is the primary access for coronary angiography and interventional therapy, and the recommended standard is IA level [20]. Furthermore, it is increasingly becoming the preferred route for coronary procedures worldwide [21]. However, the complications after TRA cannot be ignored, and the choice of the hemostasis method is significant.

The use of the radial artery hemostats device is the primary method of hemostasis after the TRA. A particular device is used to compress the puncture site of the radial artery to achieve hemostasis. These devices are convenient for patients and clinicians, reducing complications and improving patients' comfort. There are also several clinical studies on radial artery hemostatic devices. Cong et al. compared the pressure dressing, the pneumatic compression device, and the rotary compression pad device in clinical effect and found that the radial artery hemostatic device had more advantages than the pressure dressing and had apparent effects in shortening hemostasis time, reducing patient discomfort, and decreasing the incidence of RAO [9]. Due-Tønnessen et al. [22] conducted a trial to compare a new type of hemostatic device, the RY Stop, with the TR Band, and found that the incidence of RAO in the RY Stop group was 5% within 90 days. Although there was a small amount of bleeding, it was not inferior to the TR Band in the occurrence of complications and other effects. In our study, the new radial artery hemostasis device based on the patent also achieved adequate hemostasis, fully demonstrating the effectiveness of the patented hemostasis.

Regarding the safety of the radial artery hemostats device, this study found no statistically significant differences between the two groups in terms of the incidence of complications such as bleeding, ecchymosis, hematoma, skin damage, and local infection after removal of the device (*P* > 0.05), and this is also close to the findings reported in some other studies [11, 22–24]. The CD group showed a higher bleeding rate (10%) related to the definition of bleeding in this study. We counted the bleeding data, including the decompression process and after removing the hemostatic device, resulting in the high bleeding rate. However, no bleeding occurred in the CD group after removing the device in the actual observation. Although the personnel involved in decompression were trained before the study, the new radial artery hemostasis device, compared to the more commonly used TR Band, was prone to bleeding due to rapid discharge during the decompression phase or some other operational reasons.

In terms of patients' comfort, we found that the new radial artery hemostasis device demonstrated superiority in pain and swelling due to a softer and more flexible silicone wristband, which optimized the fit to the wrist and provided better hemostasis, and the design of the airbag bar, which achieved precise compression while reducing the impact on surrounding tissues. These designs had greatly improved the comfort of patients. The rigid plastic plate of the TR Band hemostatic device can easily increase the area of thrust surface, affect venous blood flow, increase swelling and numbness, and other discomforts. This study also found that the CD group and TR group had different effects on blood pressure and pulse at different time points after the operation, but the changes in blood pressure and pulse were affected by many factors, such as medication, mood, activity, and other related factors. In a study on perioperative blood pressure, Ackland et al. [25] pointed out that the influence of physiological factors on blood pressure cannot be ignored and that there was a close relationship between organ blood flow, microcirculation, and arterial pressure. At the same time, he also suggested the rationality of using mean artery pressure (MAP) instead of systolic and diastolic blood pressure. Cubero et al. [26] found in their study that the hemostatic pressure referenced by MAP could reduce the incidence of the RAO based on adequate hemostasis. In our study, the systolic blood pressure of the two groups showed some differences before the operation (*P*=0.052 > 0.05), so the blood pressure at different time points after the operation was easily affected by the primary blood pressure. Both groups of patients showed different anxiety before the operation, the blood pressure and pulse could be affected by this, and for some patients, the effect of using antihypertensive drugs should not be ignored. At the same time, considering the small sample size, we believe that the difference needs further experimental verification.

There was no significant difference in the influence of the new radial artery hemostasis device and TR Band on SpO_2_ and wrist circumference (*P* > 0.05). At 1 h and 6 h after the operation, SpO_2_ was decreased, and wrist circumference was increased compared with that before the operation because the hemostat stopped arterial bleeding and affected venous blood flow at the same time. With the gradual decrease of pressure, there was no statistically significant difference in SpO_2_ between the two groups at 24 h postoperatively compared with preoperatively (*P* > 0.05), indicating no significant difference in peripheral circulation between the two groups. The difference in wrist circumference was statistically significant (*P* < 0.05), indicating that there was still a certain degree of swelling in the wrist 24 h after operation. The use of the two hemostatic devices will affect the patients' distal blood supply and wrist circumference in a short time, but with the decompression and the removal of the wristband, these temporary effects will be restored.

### 4.1. Study Limitations

As a single-center prospective trial, this study focused on the safety of the sample of the new radial artery hemostasis device. It was a small sample of a preclinical trial study, resulting in a bias in some postoperative analysis results. Then, a meta-analysis of RAO by Rashid et al. [27] showed that RAO incidence fluctuated between 0.8% and 38%, while most patients did not assess the patencies of the radial artery before being discharged. Plethysmography and pulse oxygen saturation was used to evaluate RAO preliminarily for this study. However, due to the dual blood supply of the hand, it is highly likely that RAO could not be found in time, and vascular ultrasound should still be perfected in the follow-up period to obtain a more accurate evaluation. Finally, there was no blindness in our study. Relevant operators and patients knew different devices for hemostasis, and some patients expressed concerns about the efficacy of the new radial compression device, which could impact the evaluation of some indicators. In a later large-sample, multicenter clinical studies, we should also consider refining the design protocol to compensate for these inadequacies to obtain complete and accurate trial results.

## 5. Conclusion

This study shows that the new radial artery hemostasis device based on the patent has a positive clinical effect, achieving effective hemostasis without increasing the incidence of complications and improving patient comfort. After large-scale clinical trials are completed, it can be used as a new product to enter the market.

## Figures and Tables

**Table 1:** Baseline characteristics between the two groups.

Variable	CD group (*n* = 30)	TR group (*n* = 30)	*χ* ^2^/*t*	*P*-value
Age	59.20 ± 9.35	56.90 ± 10.52	0.895	0.374
Male	17 (56.67)	18 (60.00)	0.069	0.793
Height, cm	167.15 ± 7.94	165.77 ± 6.92	−0.719	0.475
Weight, kg	70.73 ± 11.48	66.95 ± 11.02	−1.303	0.198
BMI, kg/m^2^	24.34 ± 3.79	25.28 ± 3.45	−0.979	0.332
Hypertension	13 (43.33)	18 (60.00)	1.669	0.196
Diabetes	3 (10.00)	5 (16.67)	0.577	0.706
Hyperlipidemia	18 (60.00)	15 (50.00)	0.606	0.436
Chronic renal failure	0 (0.00)	1 (3.30)	0.000	1.000
Family history of CAD	5 (16.67)	9 (30.00)	1.491	0.222
Smoking	12 (40.00)	14 (46.67)	0.271	0.602
Platelet count, ×10^9/L	224.70 ± 97.58	228.33 ± 51.58	−0.180	0.858
PT, s	11.78 ± 0.95	11.75 ± 1.41	0.075	0.940
APTT, s	29.16 ± 13.02	26.27 ± 5.00	1.132	0.262
PT. INR	1.00 ± 0.09	1.00 ± 0.07	−0.065	0.948
Creatinine, *μ*mol/L	68.39 ± 17.60	67.43 ± 12.77	0.242	0.810
Clopidogrel	2 (6.67)	0 (0.00)	2.069	0.492
Aspirin	3 (10.00)	5 (16.67)	0.577	0.706

*Procedure characteristics*				
Sheath size (5 French)	29 (96.67)	30 (100.00)	1.017	1.000
Heparin used during procedure, IU	3166.67 ± 647.72	3083.33 ± 323.86	0.630	0.531
Procedure time, min	25.57 ± 8.76	26.77 ± 6.25	−0.611	0.544
CAG	28 (93.33)	28 (93.33)	0.000	1.000

Values are presented as mean ± standard deviation or n (%). BMI, body mass index; PT, prothrombin time; APTT, activated partial thromboplastin time; PT. INR, prothrombin time international normalized ratio.

**Table 2:** Outcome of vascular access site complications.

Complication	CD group (*n* = 30)	TR group (*n* = 30)	*χ* ^2^	*P*-value
Bleeding	3 (10.00)	1 (3.33)	1.074	0.612
Ecchymosis	2 (6.67)	2 (6.67)	0.000	1.000
Hematoma	1 (3.33)	0 (0.00)	0.000	1.000
Skin lesion	0 (0.00)	0 (0.00)	0.000	1.000
Local infection	0 (0.00)	0 (0.00)	0.000	1.000

Values are presented as n (%).

**Table 3:** Comparison of related observation indexes at the different time between the two groups.

Variable	Time point	CD group (*n* = 30)	TR group (*n* = 30)	*t*	*P*-value
SpO_2_ (%)	0 h	95.77 ± 2.24	95.667 ± 1.971	0.184	0.855
	1 h	94.80 ± 2.43	94.63 ± 2.46	0.264	0.792
	6 h	94.80 ± 2.19	94.53 ± 2.19	0.472	0.639
	24 h	95.23 ± 2.11	95.13 ± 1.66	0.204	0.839

Wrist circumference (cm)	0 h	17.13 ± 1.16	16.87 ± 0.96	0.969	0.337
	1 h	17.79 ± 1.15	17.63 ± 1.26	0.525	0.602
	6 h	17.69 ± 1.12	17.35 ± 1.07	1.213	0.230
	24 h	17.33 ± 1.13	17.21 ± 0.95	0.458	0.648

Systolic blood pressure (mmHg)	0 h	123.60 ± 14.76	131.00 ± 14.11	−1.985	0.052
	1 h	126.57 ± 13.66	133.63 ± 12.33	−2.104	0.042
	6 h	122.60 ± 13.92	132.43 ± 13.08	−2.823	0.007
	24 h	121.40 ± 11.34	131.73 ± 13.91	−3.154	0.003

Diastolic blood pressure (mmHg)	0 h	74.00 ± 7.94	75.07 ± 8.63	−0.498	0.620
	1 h	76.27 ± 9.54	76.13 ± 9.10	0.055	0.956
	6 h	73.43 ± 10.29	78.33 ± 8.19	−2.041	0.046
	24 h	70.47 ± 7.54	77.77 ± 8.84	−3.441	0.001

Pulse	0 h	73.53 ± 8.36	69.60 ± 9.00	1.754	0.085
	1 h	74.50 ± 9.39	68.77 ± 7.57	2.604	0.012
	6 h	74.37 ± 10.31	66.20 ± 7.54	3.502	0.001
	24 h	73.90 ± 9.65	69.63 ± 8.34	1.832	0.072

Values are presented as mean ± standard deviation.

## Data Availability

All the data generated or analyzed during this study are included in this article.

## Abbreviations

CAG: Coronary angiography
PCI: Percutaneous coronary intervention
CAD: Coronary artery disease
TFA: Transfemoral access
TUA: Transulnar access
TRA: Transradial access
RAO: Radial artery occlusion
VAS: Visual analogue scale
MAP: Mean artery pressure.

## Supplementary Materials

Supplementary Materials Supplementary Figure 1: Initial sample design. Supplementary Figure 2: Different stages of sample improvement design. Supplementary Figure 3: Diagram of stress test. Supplementary File 4: Research ethics review approval.
