# Supplementary material for: Study on the Safety of the New Radial Artery Hemostasis Device
Source: J Interv Cardiol. 2022 Apr 5;2022:2345584. doi: 10.1155/2022/2345584 (PMC9005317; doi:10.1155/2022/2345584)
Supplement: Supplementary Materials — Supplementary Figure 1: Initial sample design. Supplementary Figure 2: Different stages of sample improvement design. Supplementary Figure 3: Diagram of stress test. Supplementary File 4: Research ethics review approval. [file 2345584.f1.zip › 2345584.f1/Supplementary Figure2. Different stages of sample improvement design.pdf]

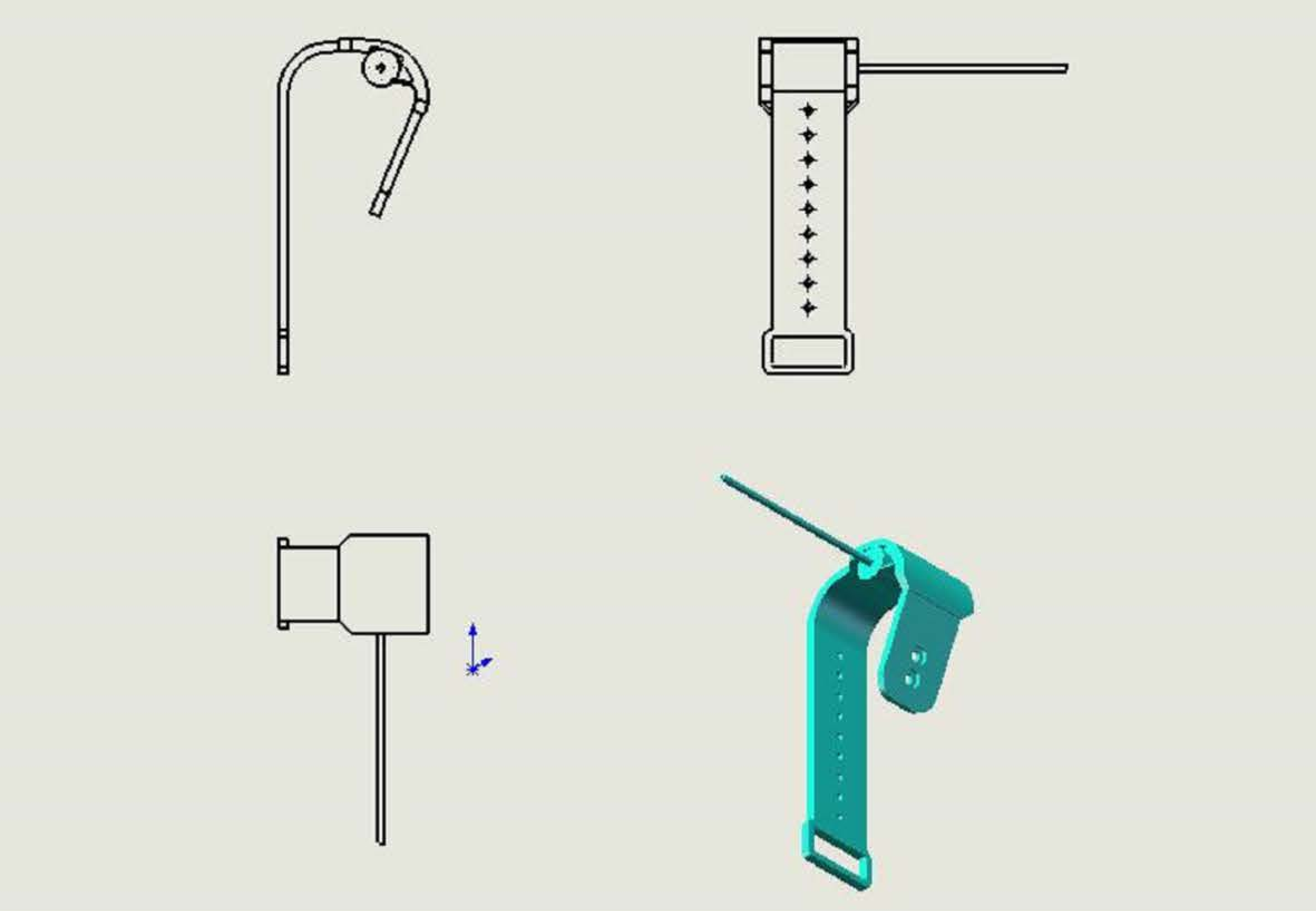

Stage1.Improved 3D design drawing based on patent

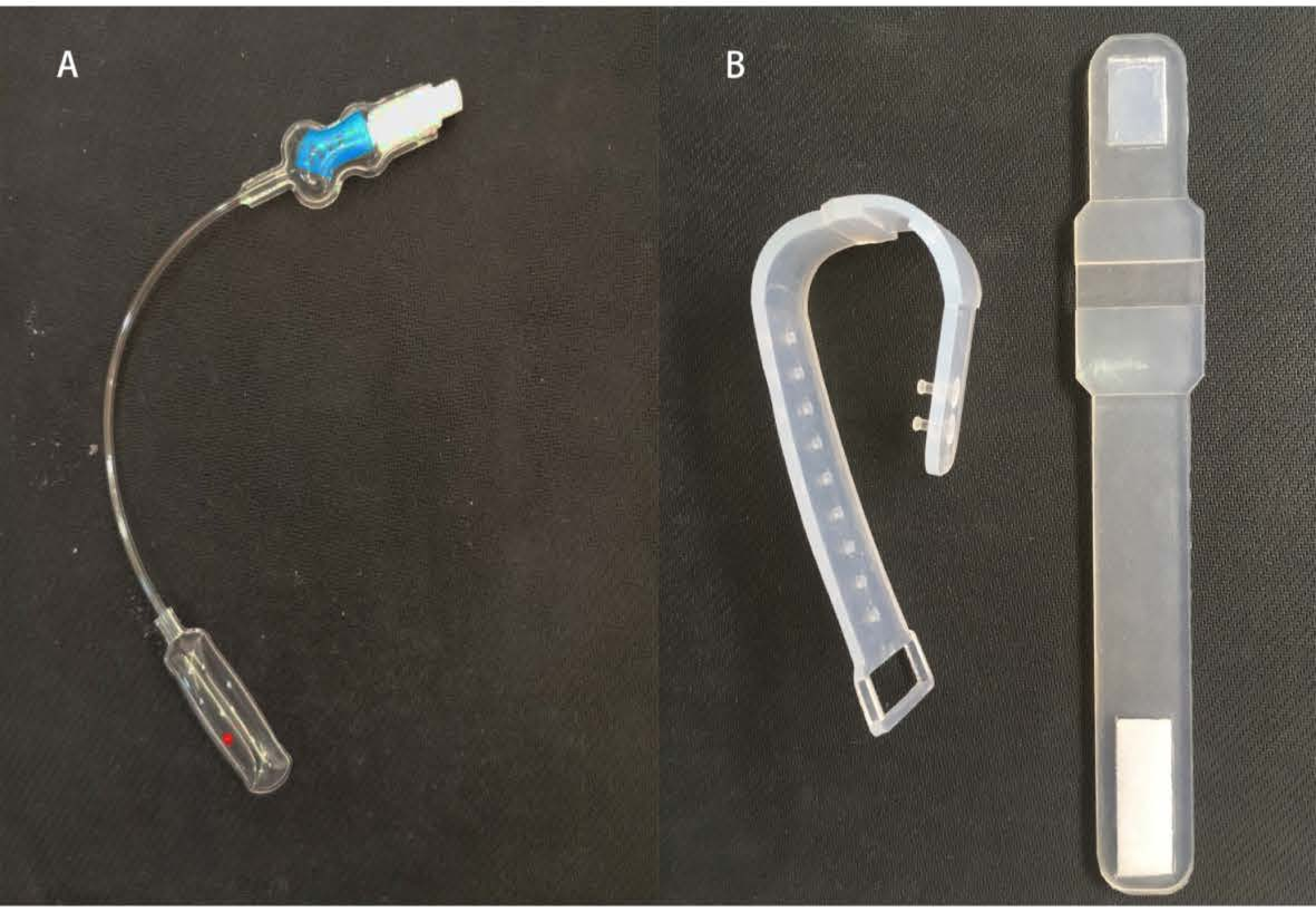

Stage2.A sparate design of the compression subject(A) and the different designs of the wristband(B).

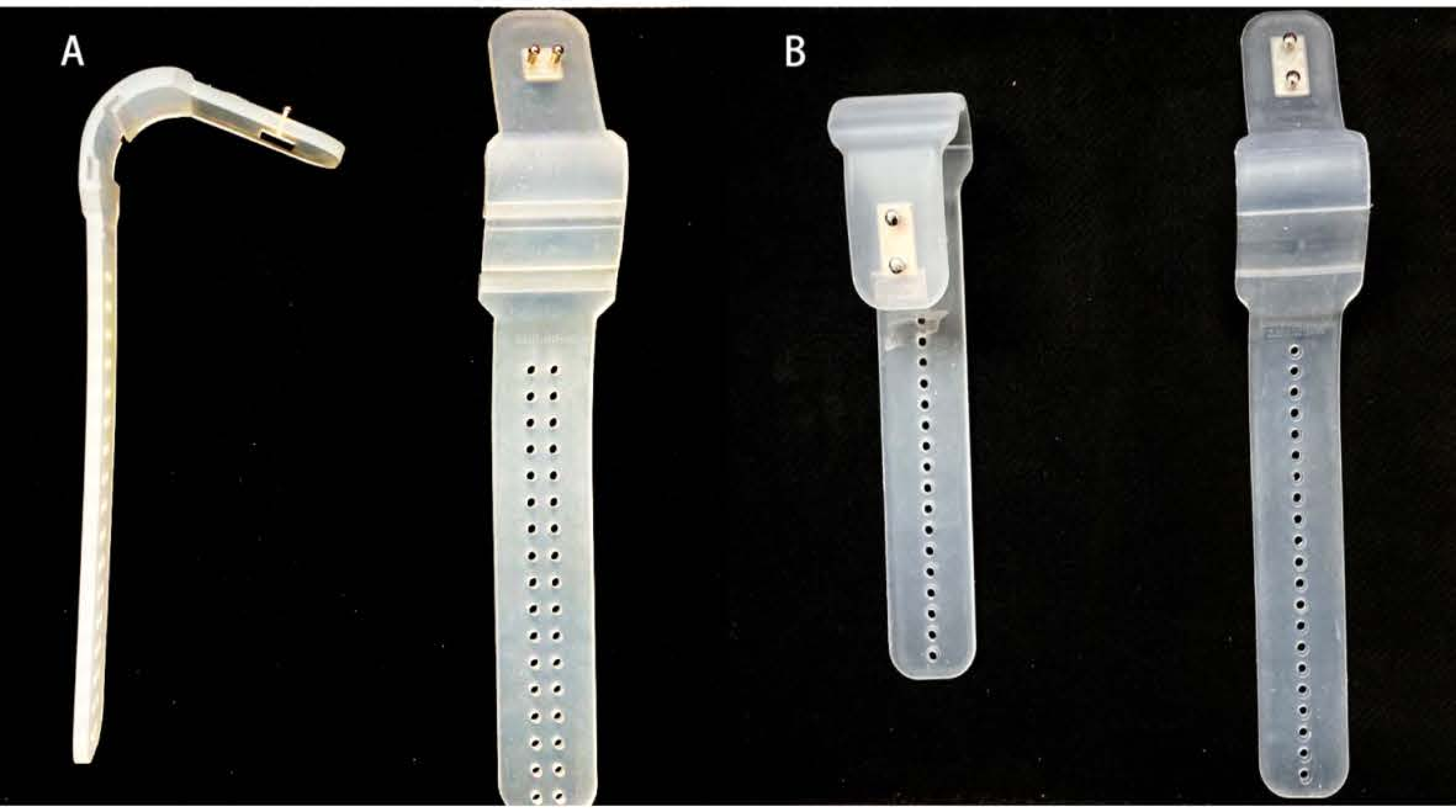

Stage3.Two types of wristbands with buckle design.Horizonal(A) and vertical(B) bulges and matching holes.
