# Supplementary figures and images for: Study on the Safety of the New Radial Artery Hemostasis Device
Source: J Interv Cardiol. 2022 Apr 5;2022:2345584. doi: 10.1155/2022/2345584 (PMC9005317; doi:10.1155/2022/2345584)

A

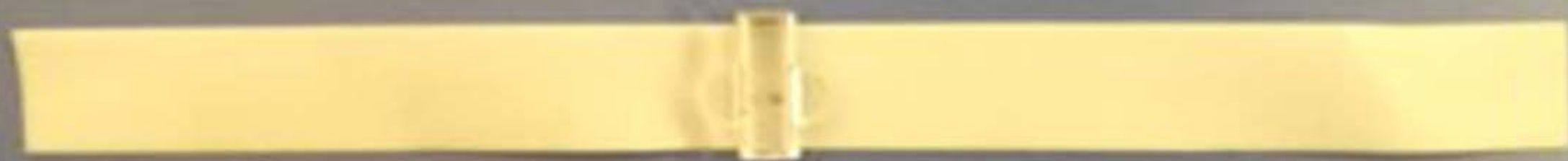

B

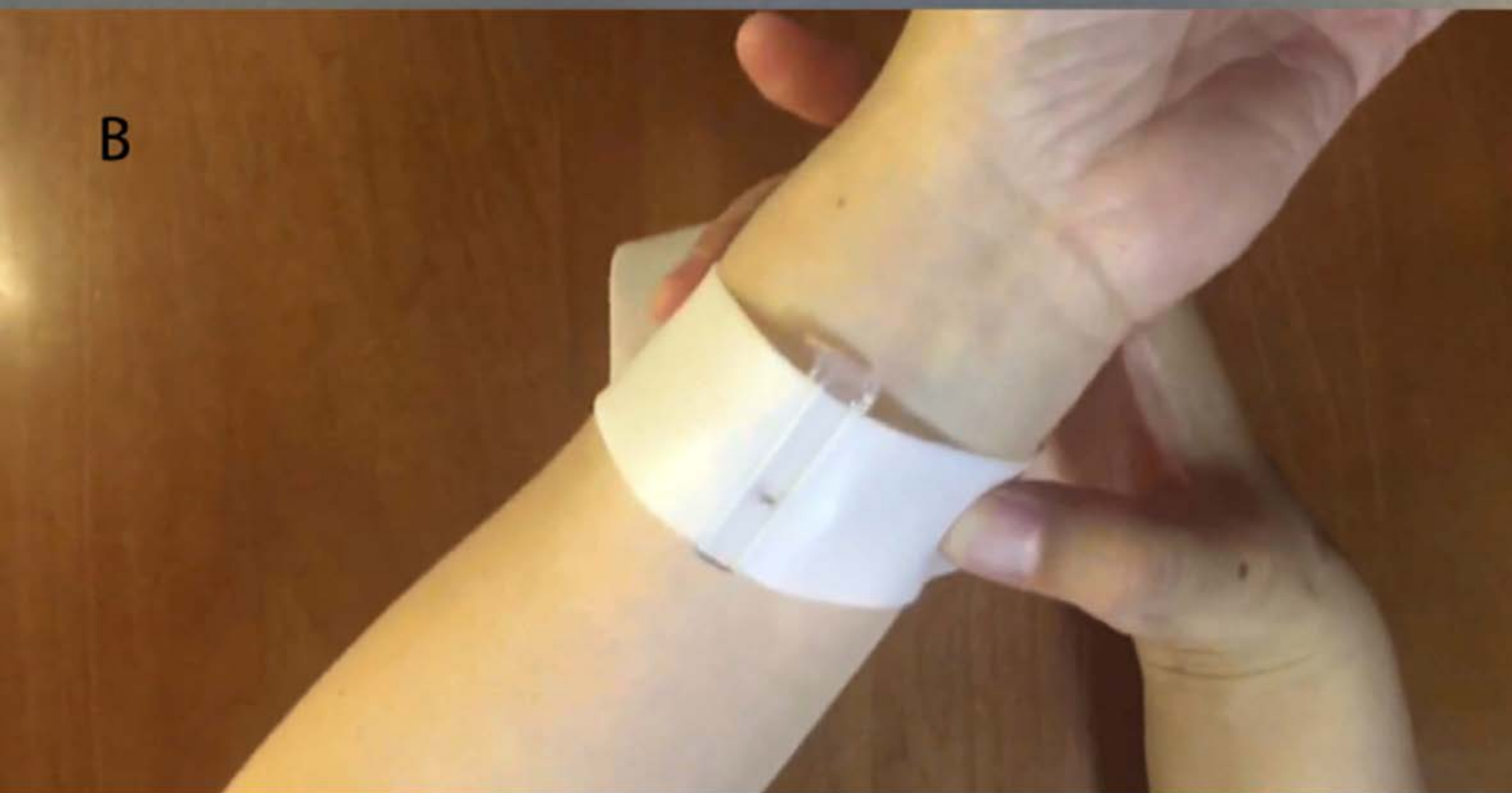

A. The model of sample      B. Operation diagram

Supplement: Supplementary Materials — Supplementary Figure 1: Initial sample design. Supplementary Figure 2: Different stages of sample improvement design. Supplementary Figure 3: Diagram of stress test. Supplementary File 4: Research ethics review approval. [file 2345584.f1.zip › 2345584.f1/Supplementary Figure1. Initial sample design.pdf]

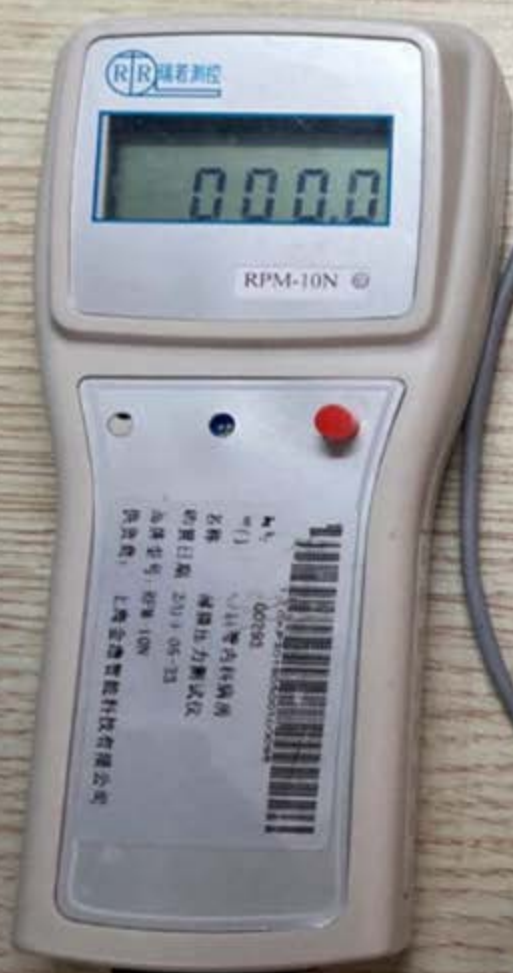

Supplement: Supplementary Materials — Supplementary Figure 1: Initial sample design. Supplementary Figure 2: Different stages of sample improvement design. Supplementary Figure 3: Diagram of stress test. Supplementary File 4: Research ethics review approval. [file 2345584.f1.zip › 2345584.f1/Supplementary Figure3. Diagram of stress test.pdf]

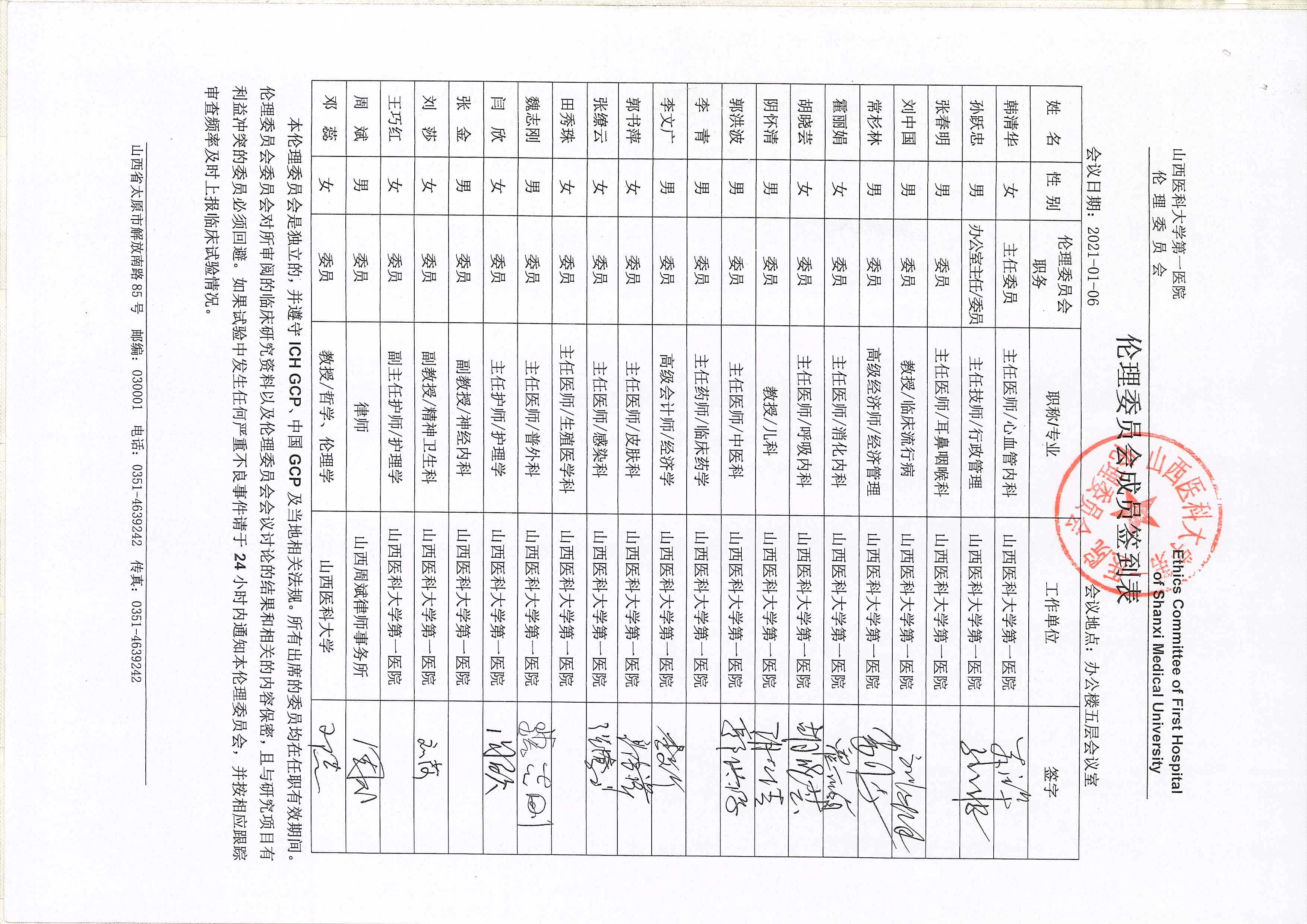

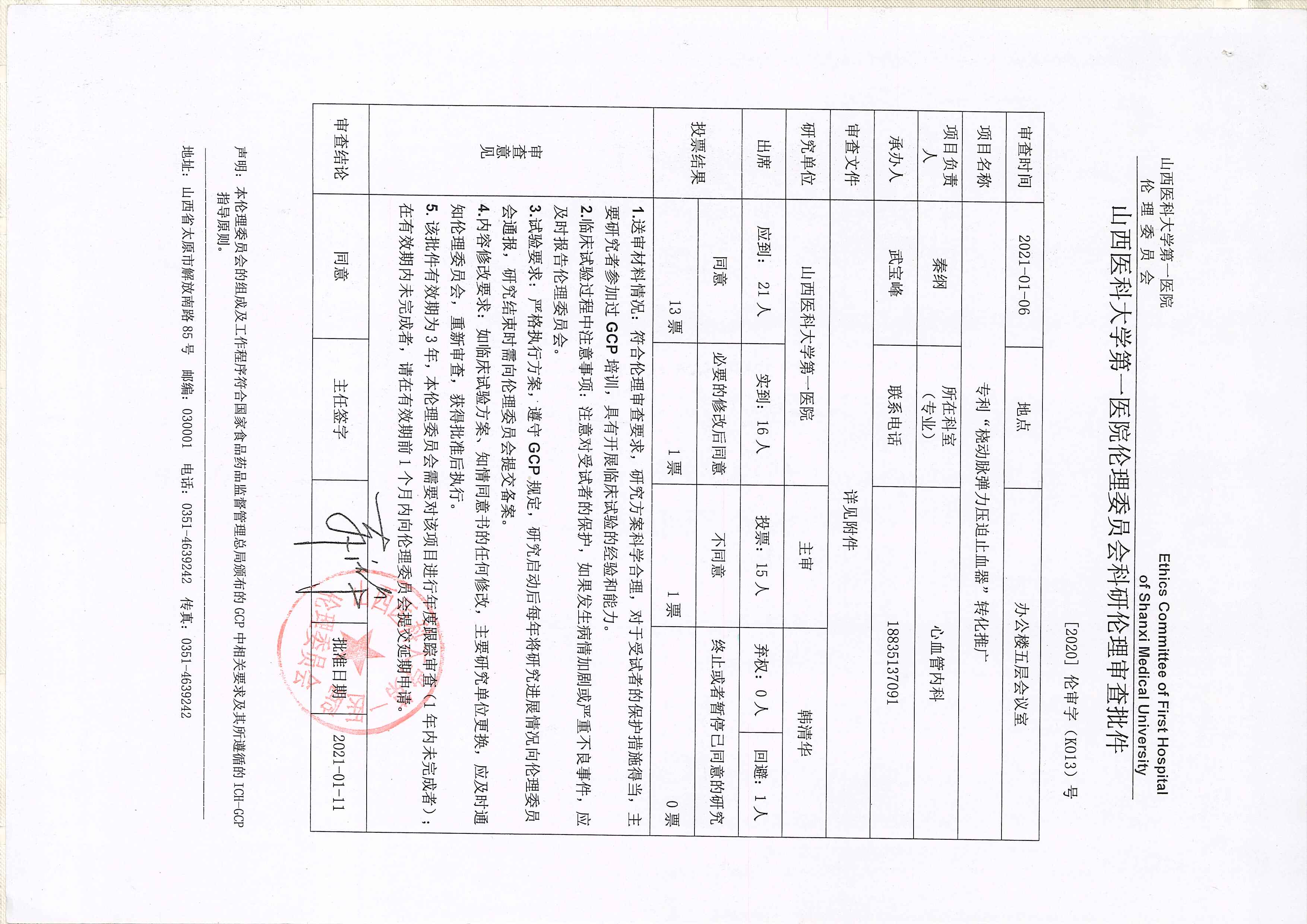

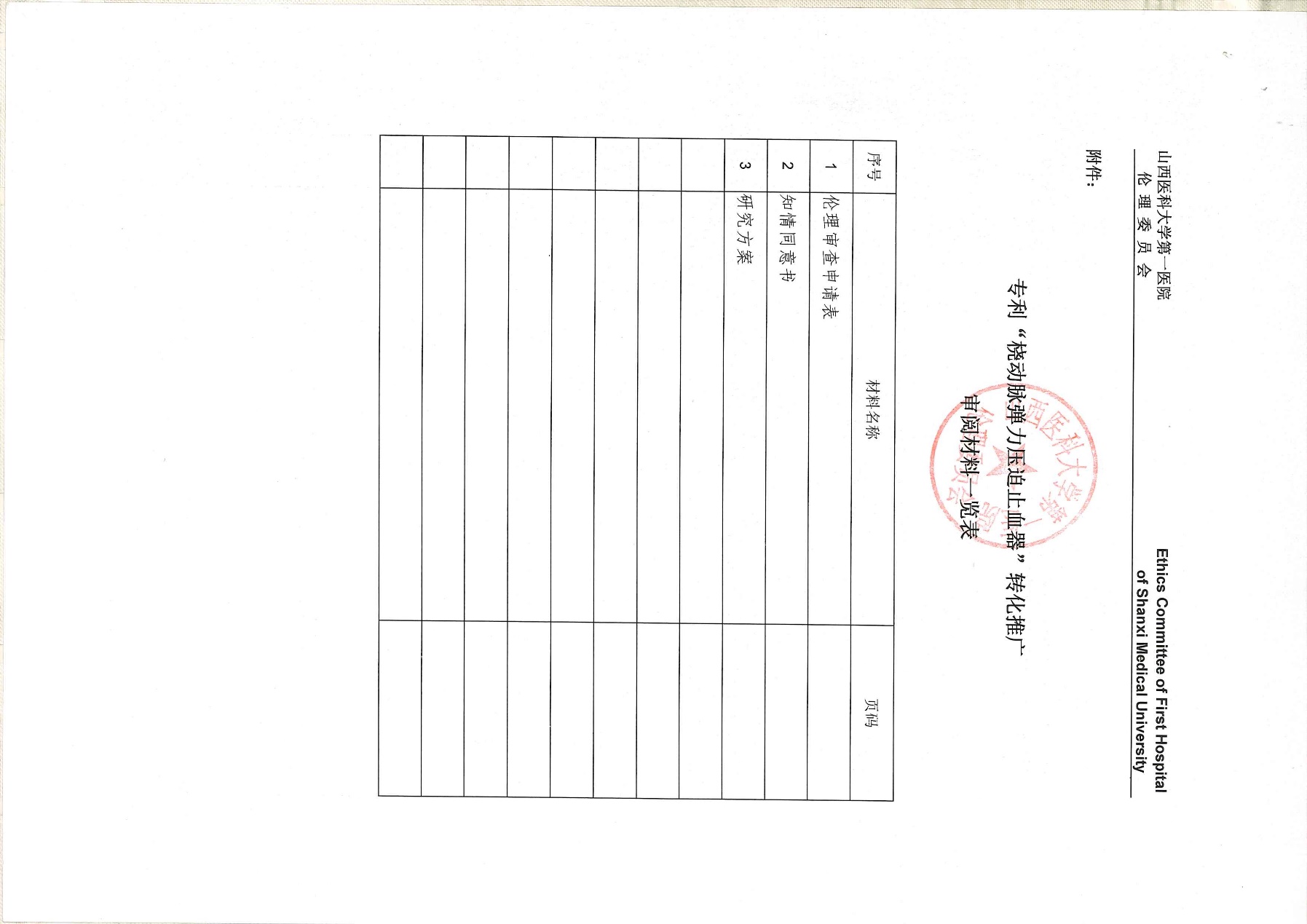

Supplement: Supplementary Materials — Supplementary Figure 1: Initial sample design. Supplementary Figure 2: Different stages of sample improvement design. Supplementary Figure 3: Diagram of stress test. Supplementary File 4: Research ethics review approval. [file 2345584.f1.zip › 2345584.f1/Supplementary File 4. Research Ethics Review Approval.docx]
